# Association between tea and coffee consumption and brain cancer risk: an updated meta-analysis

**DOI:** 10.1186/s12957-019-1591-y

**Published:** 2019-03-15

**Authors:** Yang Song, Zhiyun Wang, Yanyu Jin, Jie Guo

**Affiliations:** 1 Department of Neurology, Tianjin First Center Hospital, No. 24, Fukang Road, Nankai District, Tianjin, 300192 China; 2 Department of General Surgery, Tianjin First Center Hospital, Tianjin, 300192 China

**Keywords:** Coffee, Tea, Brain cancer, Glioma, Meta-analysis

## Abstract

**Background:**

Previous studies had demonstrated some associations between coffee and tea consumption and brain cancer risk resulted in an inconsistent relationship. We therefore performed this study to further explore the association between them.

**Method:**

By searching PubMed, Embase, and Web of Science, we retrieved up to 1 November 2018, 11 relevant literature of publications were collected by 2 people eventually. Stata 14.0 software was used for data analysis.

**Results:**

In total, 11 articles (11 articles for coffee, 8 articles for tea, and 4 articles for coffee plus tea) were used in this meta-analysis. A statistically significant protective effect of coffee consumption and brain cancer risk was found (RR = 0.785, 95% CI = 0.580–0.984, *I*^2^ = 65.6%, *P*_for heterogeneity_ = 0.001), especially in Asian populations (RR = 0.217, 95% CI = 0.042–0.896). However, the association between the risk of brain cancer and tea consumption was non-significant in the whole result (RR = 0.897, 95% CI = 0.739–1.088, *I*^2^ = 29.9%, *P*_for heterogeneity_ = 0.189), but significant in American populations (RR = 0.798, 95% CI = 0.646–0.986). Interestingly, the RR was 0.684 (95% CI = 0.481–0.975) for the risk of brain cancer when compared the highest versus the lowest category consumption of coffee plus tea.

**Conclusion:**

Findings from this study suggested that higher consumption of coffee may contribute to the lower development of brain cancer in Asian populations. Tea consumption had an inverse association for the risk of brain cancer in American populations, instead of other populations.

**Electronic supplementary material:**

The online version of this article (10.1186/s12957-019-1591-y) contains supplementary material, which is available to authorized users.

## Introduction

Brain cancer is one of the most common malignant tumors in adult primary intracranial tumors [1, 2]. The role of several nutritional factors in the prevention of brain cancer has been investigated. For example, consumption of fresh fruits and vegetables [3], fresh fish [4], and vitamin supplements [5–7] have been acted preventive agents. Coffee and tea are popular with people as two kinds of drinks. Coffee consumption had been linked to some cancers. Horisaki et al. performed a dose-response meta-analysis indicated that coffee consumption may be weakly inversely associated with the risk of colorectal cancer in Japanese [8]. Previous studies published by Lukic et al. [9] and Lafranconi et al. [10] suggested that a prospective effect of coffee consumption on the risk of endometrial cancer. Highest versus lowest category of tea consumption had been confirmed of decreased with liver cancer [11, 12], ovarian cancer [13], gastric cancer [14], oral cancer [15, 16], and endometrial cancer [17].

A previous meta-analysis was published to explore the association between coffee and tea consumption and the risk of glioma in adults [18]. They concluded that the highest versus lowest category of coffee consumption, tea consumption, and coffee plus tea consumption had no effect on the risk of brain cancer. However, many studies had been published to further assess the association between coffee and tea consumption and the risk of brain cancer since that meta-analysis. For this reason, this paper increased the sample size and improved the test efficiency through an updated meta-analysis to obtain more authentic and reliable analysis results, which is helpful to clarify whether coffee or tea consumption has some inverse effects on brain cancer development, and finally provides evidence of prevention for brain cancer.

## Methods

### Search strategy and inclusion criteria

Two authors independently checked the databases of PubMed, Embase, and Web of Science to find the relevant articles which investigated the association between tea and/or coffee consumption and risk of brain cancer from inception up to 1 November 2018. The following search terms were used: “tea” OR “coffee” OR “drinking” combined with “brain cancer” OR “brain tumor” OR “glioma.” To identify titles and abstracts of relevant literature, reference lists of studies were checked manually.

All studies included in this meta-analysis should meet the following criteria: (1) assessment of the tea and/or coffee consumption and brain cancer or glioma risk; (2) observational studies; (3) the detailed data of studies must be completely provided in all participants directly or indirectly; (4) human studies.

### Data extraction

Data were extracted independently by two researchers (YS and ZYW). All the information listed in Table 1 in each study was abstracted carefully. The disagreements with these two researchers were resolved by discussion and consensus.

**Table 1:** Characteristics of the included studies on tea and coffee consumption and brain cancer risk

Study, year	Country	Study design	Participants (cases)	Age (years)	Outcome	Exposure and category	RR (95% CI)	Adjustment for covariates
Baglietto et al. 2011	Australia	Cohort	39,766 (67)	27–81	Brain glioma	Coffee: 4 cups/day or more vs. < 1cup /day	0.51 (0.20–1.13)	Adjusted for sex, country of birth, total energy intake from diet, and level of education.
Burch et al. 1987	Canada	HBCC	475 (247)	25–80	Brain cancer	Coffee: ever vs. never Tea: ever vs. never	Coffee: 1.26 (0.76–2.09) Tea: 1.13 (0.73–1.72)	Adjusted for age, sex, area of residence, marital status, and date of diagnosis or death.
Dubrow et al. 2012	USA	Cohort	545,771 (904)	50–71	Brain glioma	Coffee: ≥6 cups/day vs. none Tea: > 3 cup/day vs. none Tea plus coffee: > 5 cup/day vs. none	Coffee: 1.04 (0.70–1.55) Tea: 0.75 (0.57–1.00) Tea plus coffee: 0.68 (0.46–1.03)	Adjusted for age, sex, race/ethnicity, height, and intake of total energy, fruit, vegetable, and nitrites.
Efird et al. 2004	USA	Cohort	133,811 (130)	≥ 25	Brain glioma	Coffee: ≥ 7 cups/day vs. < 1 cup/day	1.7 (0.8–3.6)	Adjusted for age, sex, race, smoking, education, and alcohol.
Hashibe et al. 2015	USA	Cohort	97,334 (103)	55–74	Brain glioma	Coffee: ≥ 2 cups/day vs. < 1 cup/day Tea: ≥ 1 cup/day vs. < 1 cup/day	Coffee: 0.76 (0.50–1.17) Tea: 1.04 (0.65–1.66)	Adjusted for age (continuous), sex, race, and education.
Hochberg et al. 1990	USA	PBCC	288 (160)	15–81	Brain glioma	Coffee: ≥ 4 cups/day vs. < 1 cup/day	0.9 (0.5–1.8)	Adjusted for age, sex, and socio-economic status.
Holick et al. 2010	USA	Cohort	230,655 (335)	25–75	Brain glioma	Coffee: ≥ 4 cups/day vs. none Tea: ≥ 8 cups/day vs. none Tea plus coffee: ≥ 5 cups/ day vs. < 1 cup/day	Coffee: 0.80 (0.54–1.17) Tea: 0.71 (0.45–1.12) Tea plus coffee: 0.60 (0.41–0.87)	Adjusted for age, sex, total caloric intake. (Further adjustments for cigarette smoking, current smoking, intake of processed meat, alcohol, fruit and vegetables , and, for women, reproductive factors did not change the risk estimates)
Malmir et al. 2017	Iran	HBCC	384 (128)	43.4 ± 14.6	Brain glioma	Coffee: T3 vs. T1 Tea: T3 vs. T1 Tea plus coffee: T3 vs. T1	Coffee: 0.09 (0.03–0.24) Tea: 0.33 (0.13–0.86) Tea plus coffee: 0.35 (0.15–0.83)	Adjusted for energy intake, physical activity, family history of cancers, family history of glioma, marital status, education, highrisk occupation, high-risk residential area, duration of cell phone use, supplement use, history of exposure to the radiographic X-ray, history of head trauma, history of allergy, history of hypertension, smoking status, exposure to chemicals, drug use, personal hair dye, frequent fried food intake, frequent use of barbecue, canned foods and microwave, meats and processed meats, legumes and nuts, fruits, salt and interaction effects of tea and coffee consumption, and BMI.
Michaud et al. 2010	France, Italy, Spain, Denmark, Germany, Greece, Netherlands, Norway, Sweden, UK	Cohort	521,448 (343)	25–70	Brain glioma	Coffee: Q5 vs. Q1 Tea: Q4 vs. Q1 Tea plus coffee: Q5 vs. Q1	Coffee: 0.98 (0.67–1.41) Tea: 1.05 (0.75–1.48) Tea plus coffee: 1.02 (0.72–1.44)	Adjusted for age, sex, country, body mass index, smoking status, and education.
Nelson et al. 2012	USA	Cohort	8006 (9)	45–68	Brain glioma	Coffee: ≥ 4 cups/day vs. none Tea: ≥ 4 cups/day vs. none	Coffee: 0.89 (0.08–10.02) Tea: 1.21 (0.22–6.76)	Adjusted for age, education, and triceps skinfold thickness
Ogawa et al. 2016	Japan	Cohort	106,324 (157)	40–69	Brain cancer Brain glioma	Coffee: ≥ 3 cups/day vs. ≤ 4 days/week Tea: ≥ 3 cups/day vs. ≤ 4 days/week	Brain cancer: Coffee: 0.48 (0.23–1.00) Tea: 0.55 (0.17–1.84) Brain glioma: Coffee: 1.07 (0.70–1.62) Tea: 1.05 (0.54–2.05)	Adjusted for age, sex, BMI, pack-years of cigarettes (never and past, 0–20, > 20), alcohol intake (non and past and 1–3 times/month, drinker ≤ 150, 150 g of ethanol per week), green tea (≤ 4 days/week, 1–2 cups/day, ≥ 3 cups/day), and past history of allergy, past history of diabetes mellitus.

Abbreviations: *RR* relative risk, *CI* confidence interval, *PBCC* population-based case-control studies, *HBCC* hospital-based case-control studies, *T3* tertile 3, *T1* tertile 1, *Q1* quartile 1, *Q4* quartile 4, *Q5* quartile 5

### Statistical analysis

Stata 14.0 software was used for data analysis. The combined relative risk (RR) with its 95% confidence interval (CI) for tea and coffee consumption and brain cancer risk were calculated. Forest plots for the association between tea and coffee consumption and brain cancer risk were mapping. Heterogeneity between studies was evaluated by chi-square-based *Q* test and *I*^2^ test [19, 20]. The random-effects model was used for all the analysis. Potential publication biases were examined using Egger’s linear regression [21] and Begg’s funnel plots [22]. Sensitivity analysis was done to estimate the stability of the results by removing a single study from the analysis. Subgroup analyses by study design and geographic location were also performed. *P* < 0.05 was considered statistically significant.

## Results

### Study selection and study characterization

**Fig. 1:**
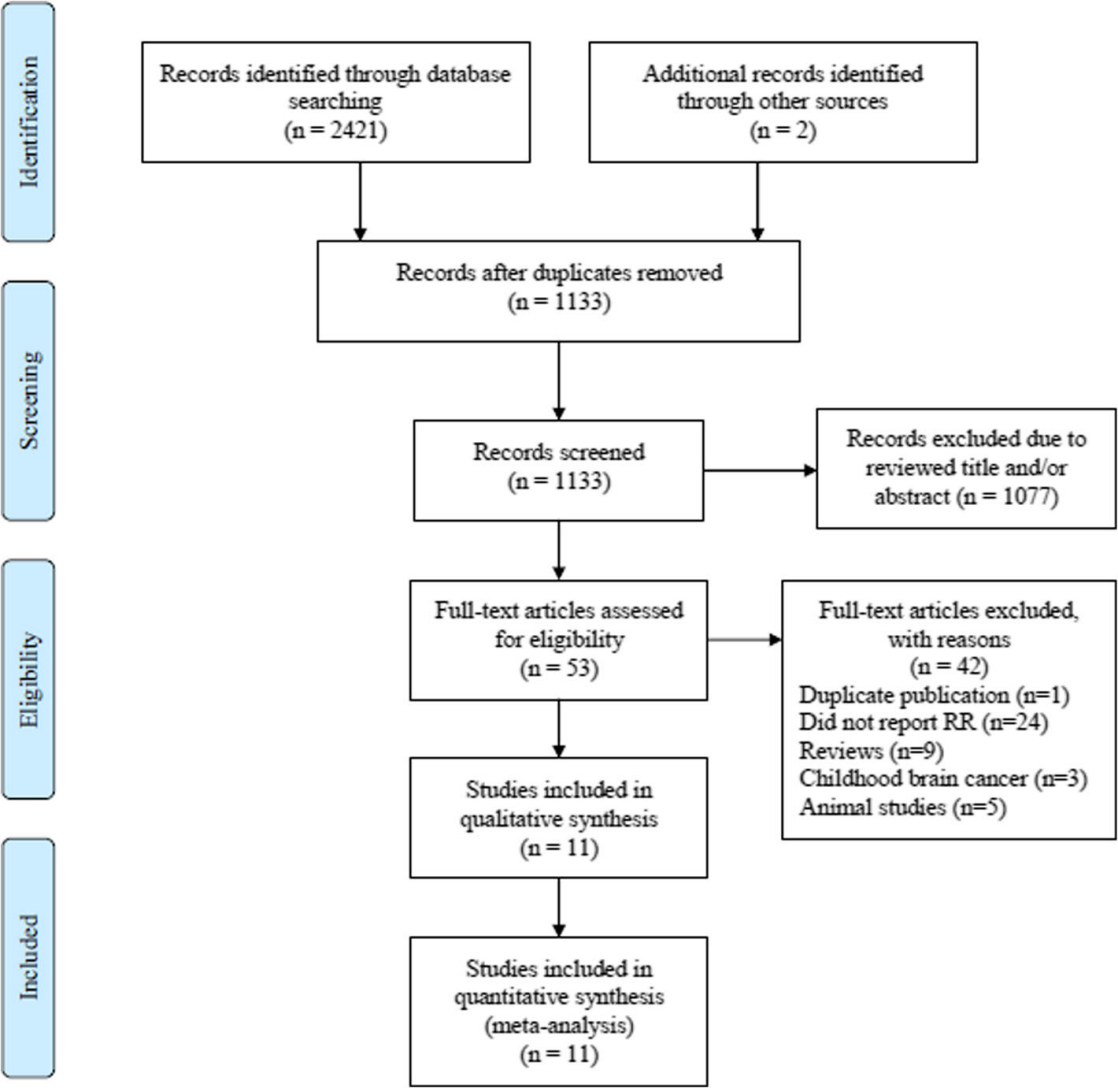
Flow chart of meta-analysis for exclusion/inclusion of studies

According to retrieval methods above, 2421 relevant studies from the databases (671 articles from PubMed, 1299 articles from Web of Science, and 451 articles from Embase) and 2 additional records identified when we reviewed the discussion and introduction of the included studies were selected. In total, 11 articles [23–33] were included in this study, as shown in Fig. 1. Six studies were from the USA, 1 from Australia, 1 from Canada, 1 from Iran, 1 from Japan, and 1 from Europe (France, Italy, Spain, Denmark, Germany, Greece, Netherlands, Norway, Sweden, and the UK). Eight of the 11 included studies were cohort design, and the remaining 3 studies were case-control design. The characteristics of the observational studies are shown in Table 1.

### Coffee consumption and the risk of brain cancer

**Fig. 2:**
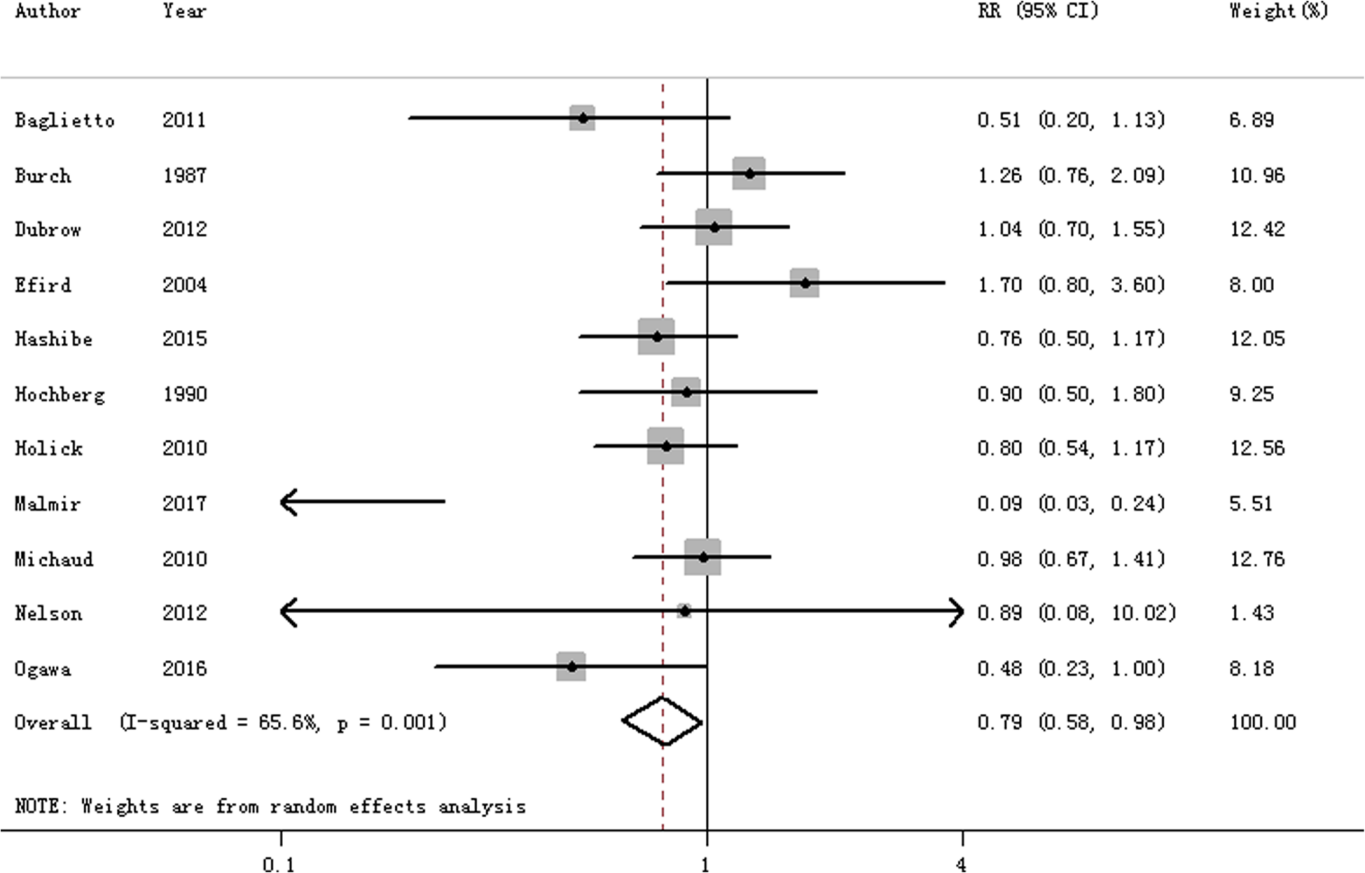
The forest plot of the relationship between coffee consumption and the risk of brain cancer

All of the 11 included studies were used to assess the association between coffee consumption and brain cancer risk. Pooled RR suggested that highest category of coffee consumption could reduce the risk of brain cancer (RR = 0.785, 95% CI = 0.580–0.984, *I*^2^ = 65.6%, *P*_for heterogeneity_ = 0.001) (Fig. 2), when compared with the lowest category. Ten of the 11 included studies were performed about coffee consumption and glioma risk, resulting in a statistically significant protective effect of coffee consumption on the risk of glioma (RR = 0.760, 95% CI = 0.548–0.972). This result is showing a statistically significant protective effect of coffee consumption in the cohort studies (RR = 0.858, 95% CI = 0.700–0.992), instead of case-control studies. Furthermore, we also explored whether the risk of brain cancer was related to geographic location. The results from our analysis indicated that an inverse association was only found in Asian populations (RR = 0.217, 95% CI = 0.042–0.896), but not in other populations else. Table 2 shows the results for both whole and subgroup analyses.

**Table 2:** Summary risk estimates of the association between tea and coffee consumption and brain cancer risk

Subgroups	Tea consumption (highest vs. lowest category)				Coffee consumption (highest vs. lowest category)			
	Studies, *n*	RR (95% CI)	*I*^*2*^ (%)	*P*_heterogeneity_	Studies, *n*	RR (95% CI)	*I*^*2*^ (%)	*P*_heterogeneity_
All studies	8	0.897 (0.739–1.088)	29.9	0.189	11	0.785 (0.580–0.984)	65.6	0.001
Glioma	7	0.846 (0.683–1.047)	24.6	0.241	10	0.760 (0.548–0.972)	63.9	0.003
Cohort	6	0.891 (0.755–1.051)	0.0	0.471	8	0.858 (0.700–0.992)	20.1	0.270
Case-control	2	0.658 (0.199–2.179)	81.5	0.020	3	0.507 (0.142–1.810)	90.1	< 0.001
America	4	0.798 (0.646–0.986)	0.0	0.595	6	0.912 (0.740–1.124)	0.0	0.520
Europe	2	1.080 (0.828–1.410)	0.0	0.792	2	1.070 (0.793–1.444)	0.0	0.433
Asia	2	0.643 (0.205–2.016)	79.9	0.026	2	0.217 (0.042–0.896)	84.9	0.010
Oceania	–	–	–	–	1	–	–	–

Based on Egger’s test (*P* = 0.214) and funnel plot (Additional file 1: Figure S1), there existed no publication bias. Sensitivity analysis was done, and the pooled RR ranged from 0.738 (95% CI = 0.542–0.961) to 0.905 (95% CI = 0.754–1.088).

### Tea consumption and the risk of brain cancer

**Fig. 3:**
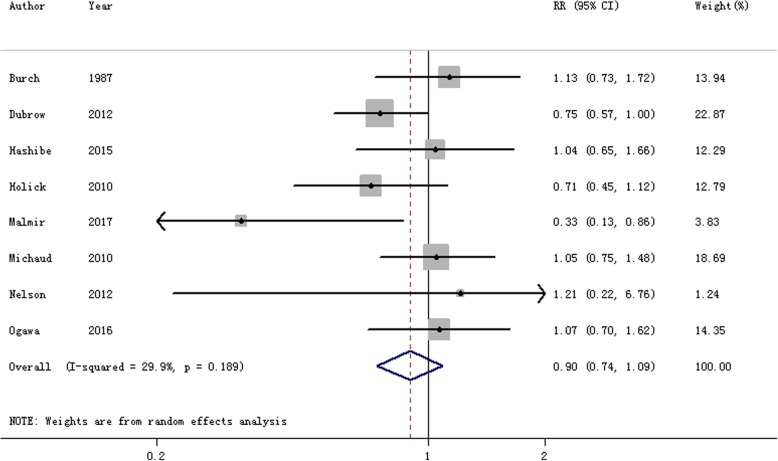
The forest plot of the relationship between tea consumption and brain cancer risk

Eight articles [24, 25, 27, 29–33] were included to explore the relationship between tea consumption and brain cancer risk. Our meta-analysis result showed that there was a non-significant association between tea consumption and brain cancer risk (RR = 0.897, 95% CI = 0.739–1.088, *I*^2^ = 29.9%, *P*_for heterogeneity_ = 0.189) (Fig. 3). Seven of the eight included studies were about tea consumption and glioma risk. The result was consistent with the brain cancer (RR = 0.846, 95% CI = 0.683–1.047). We conducted a subgroup analysis by study design (cohort studies and case-control studies). The association was non-significant in prospective studies or in case-control studies. However, when we further explored the association between brain cancer and geographic location, we found a statistically significant protective effect of tea consumption and the risk of brain cancer in American populations (RR = 0.798, 95% CI = 0.646–0.986), instead of other populations. Detailed results are shown in Table 2.

Based on Egger’s test (*P* = 0.735) and funnel plot (Additional file 2: Figure S2), there existed no publication bias between tea consumption and brain cancer risk. Sensitivity analysis was done and the pooled RR ranged from 0.863 (95% CI = 0.699–1.067) to 0.947 (95% CI = 0.763–1.176).

### Coffee plus tea consumption and the risk of brain cancer

**Fig. 4:**
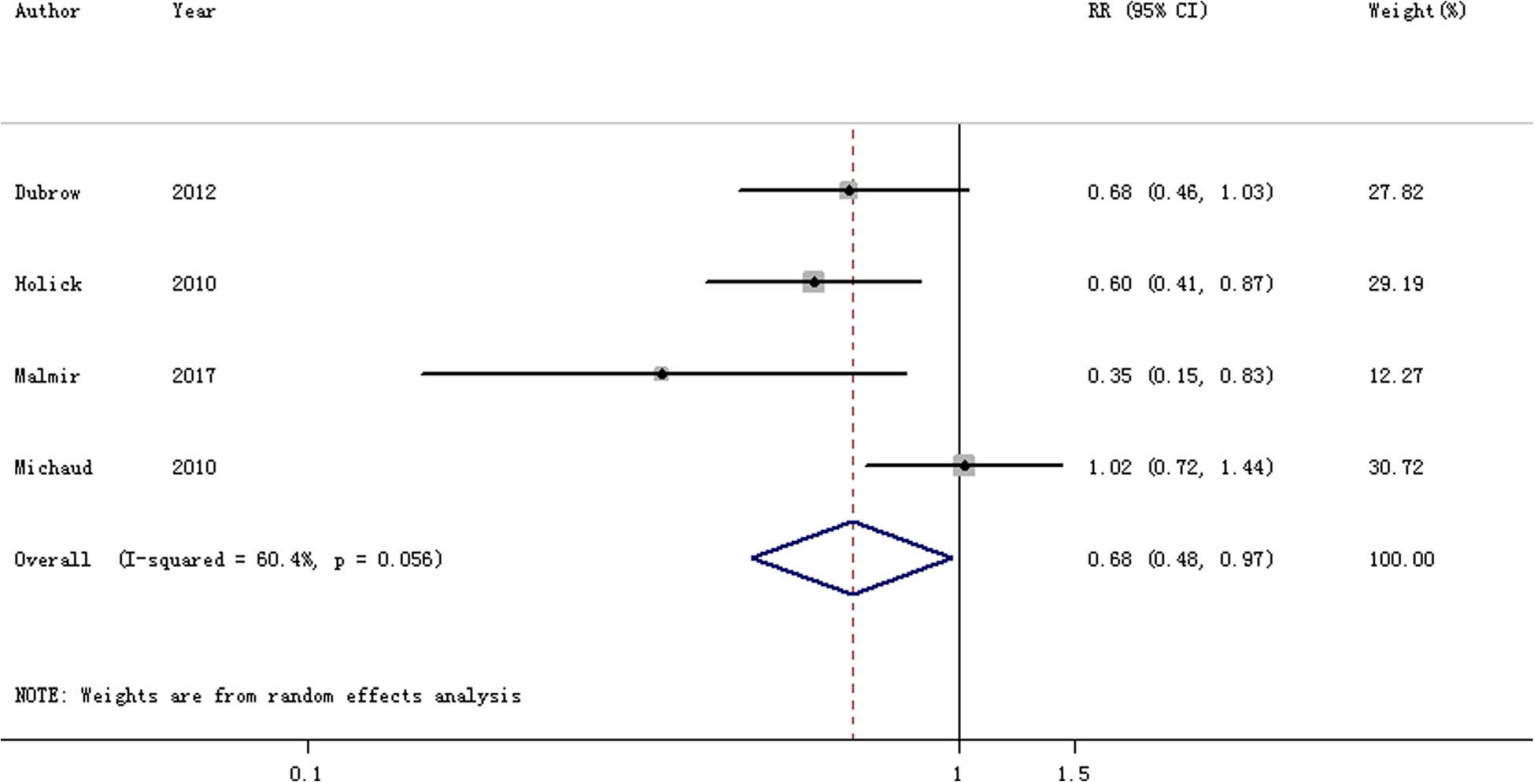
The forest plot of the relationship between coffee plus tea consumption and brain cancer risk

Four studies [25, 29–31] were performed to assess the association between coffee plus tea consumption and the risk of brain cancer. Figure 4 showed that the RR was 0.684 (95% CI = 0.481–0.975) when compared the highest versus the lowest category consumption of coffee plus tea.

## Discussion

This meta-analysis included 11 publications involving 2583 cases and 1,684,262 participants to assess the association of coffee consumption, tea consumption, and coffee plus tea consumption on the risk of brain cancer. We also performed subgroup analyses by study design and geographic location to further explore the association between coffee and tea consumption and the risk of brain cancer.

There were some differences between our meta-analysis and the study by Malerba et al. [18]. First, Malerba et al. included six studies for coffee consumption, four studies for tea consumption, three studies for coffee plus tea consumption to assess the risk of adult glioma. However, we included 11 studies for coffee consumption, 8 studies for tea consumption, and 4 studies for coffee plus tea consumption, which was more than Malerba et al. Second, the results in our analysis suggested the highest versus the lowest categories coffee consumption and coffee plus tea consumption were inversely associated with the risk of brain cancer, which was different from the results of Malerba et al. However, we got a consistent result for tea consumption and the risk of brain cancer with them.

Tea and coffee are rich in polyphenols including phenolic acids and flavonoids [34], which are known for their antioxidant activity, regulation of heterogeneous metabolite enzymes, and inhibition of tumor promotion [35], and have been shown to prevent cancer [36, 37]. Furthermore, diterpenes and caffeic acid in coffee could protect against cancer [38]. However, different tea consumed (such as green tea or black tea) and different coffee brewing method may vary on the effect of cancer [39]. We did not analyze the detailed effect about the account type of tea consumed and coffee brewing method on brain cancer due to the limited information in the individual study. Therefore, further researches are warranted to explore the potential association about them.

Sensitivity analysis for coffee and tea consumption and the risk of brain cancer was performed. For coffee consumption and brain cancer risk, the association changed from being statistically significant to not statistically significant when removed the study by Malmir et al. 2017 (RR = 0.905, 95% CI = 0.754–1.088). Therefore, the result for coffee consumption and the risk of brain cancer was not stable. Future studies with large participants are warranted to further confirm this association. For tea consumption and the risk of brain cancer, the association was not significant when removed a single study one by one, with pooled RR ranged from 0.863 (95% CI = 0.699–1.067) to 0.947 (95% CI = 0.763–1.176). For coffee plus tea consumption and the risk of brain cancer, the pooled RR changed to 0.754 (95% CI = 0.543–1.045) when removed the study by Malmir et al. 2017. Thus, more relevant studies about coffee plus tea consumption and the risk of brain cancer are needed.

Some limitations existed in our meta-analysis. First, high between-study heterogeneity existed in some results. Although meta-regression analysis was performed to explore the sources of this high between-study heterogeneity, we could not find the detailed sources of heterogeneity due to stratified analysis and meta-regression analysis concerning the relationship between coffee consumption and the risk of brain cancer. On the other hand, we used a random-effects model in all the results, which had a wider CI than the fixed-effects model. Thus, random-effects could obtain a more conservative result. Second, 3 of the 11 studies were case-control design, which could lead to some recalling bias, selective bias, and so on. However, case-control studies could also explain the relationship between coffee and tea consumption and the risk of brain cancer. Third, we could not perform the subgroup analysis by sex or lifestyle due to the limited information in each individual study. Considering sex and lifestyle may be a risk factor for coffee and/or tea consumption, future studies with detailed information about sex and lifestyle are warranted to further explore the association between coffee or tea consumption and risk of brain cancer. Fourth, the range of years in coffee or tea consumption may play a different role in the risk of brain cancer due to cancer maybe consists of various stages during aging [40]. However, the information about the range of years about coffee and tea consumption in each individual study was limited.

## Conclusions

Findings from this study suggested that higher consumption of coffee may contribute to the lower development of brain cancer in Asian populations. Tea consumption had an inverse association for the risk of brain cancer in American populations, instead of other populations. As some limitations existed in our study, future studies with detailed information about sex, lifestyle, and some other related factors are warranted to further explore the association between coffee or tea consumption and risk of brain cancer.

## Additional files

Additional file 1: **Figure S1.** Funnel plot for the analysis of publication bias between coffee consumption and brain cancer risk. (TIF 40 kb)

Additional file 2: **Figure S2.** Funnel plot for the analysis of publication bias between tea consumption and brain cancer risk. (TIF 39 kb)

## Abbreviations

CI: Confidence interval
OR: Odds ratio
RR: Relative risk
