# Supplementary figures and images for: Association between tea and coffee consumption and brain cancer risk: an updated meta-analysis
Source: World J Surg Oncol. 2019 Mar 15;17:51. doi: 10.1186/s12957-019-1591-y (PMC6419842; doi:10.1186/s12957-019-1591-y)

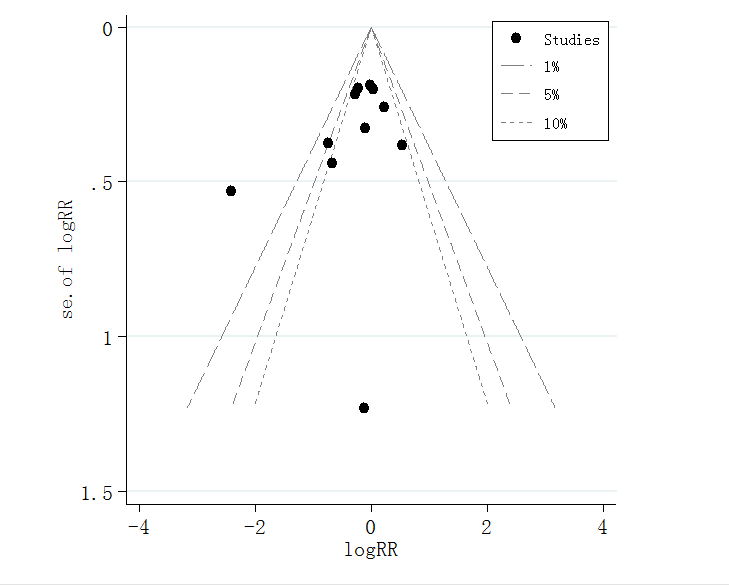

Supplement: Supplementary file 1 — Figure S1. Funnel plot for the analysis of publication bias between coffee consumption and brain cancer risk. (TIF 40 kb) [file 12957_2019_1591_MOESM1_ESM.tif]

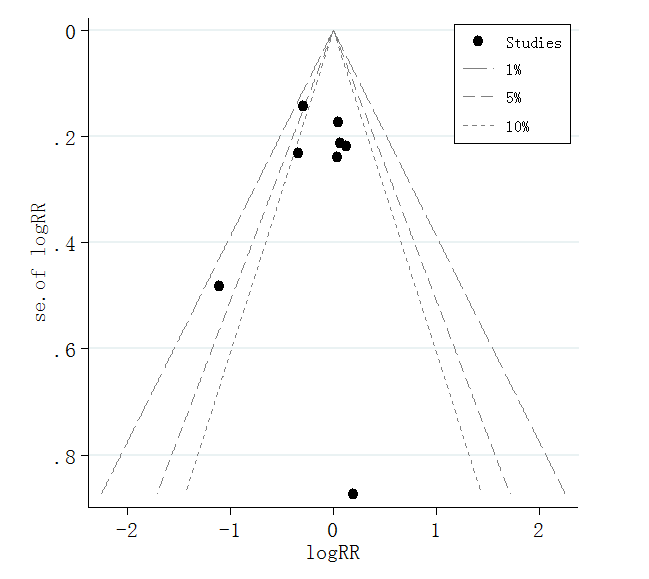

Supplement: Supplementary file 2 — Figure S2. Funnel plot for the analysis of publication bias between tea consumption and brain cancer risk. (TIF 39 kb) [file 12957_2019_1591_MOESM2_ESM.tif]
